# Role of Activins in Hepcidin Regulation during Malaria

**DOI:** 10.1128/IAI.00191-17

**Published:** 2017-11-17

**Authors:** Natasha Spottiswoode, Andrew E. Armitage, Andrew R. Williams, Alex J. Fyfe, Sumi Biswas, Susanne H. Hodgson, David Llewellyn, Prateek Choudhary, Simon J. Draper, Patrick E. Duffy, Hal Drakesmith

**Affiliations:** a MRC Human Immunology Unit, MRC Weatherall Institute of Molecular Medicine, University of Oxford, John Radcliffe Hospital, Oxford, United Kingdom; b Laboratory of Malaria Immunology & Vaccinology, NIAID, NIH, Bethesda, Maryland, USA; c Department of Veterinary Disease Biology, University of Copenhagen, Frederiksberg, Denmark; d The Jenner Institute, University of Oxford, Oxford, United Kingdom; University of South Florida

**Keywords:** hepcidin, iron, malaria, innate immunity

## Abstract

Epidemiological observations have linked increased host iron with malaria susceptibility, and perturbed iron handling has been hypothesized to contribute to the potentially life-threatening anemia that may accompany blood-stage malaria infection. To improve our understanding of these relationships, we examined the pathways involved in regulation of the master controller of iron metabolism, the hormone hepcidin, in malaria infection. We show that hepcidin upregulation in Plasmodium berghei murine malaria infection was accompanied by changes in expression of bone morphogenetic protein (BMP)/sons of mothers against decapentaplegic (SMAD) pathway target genes, a key pathway involved in hepcidin regulation. We therefore investigated known agonists of the BMP/SMAD pathway and found that *Bmp* gene expression was not increased in infection. In contrast, activin B, which can signal through the BMP/SMAD pathway and has been associated with increased hepcidin during inflammation, was upregulated in the livers of Plasmodium berghei-infected mice; hepatic activin B was also upregulated at peak parasitemia during infection with Plasmodium chabaudi. Concentrations of the closely related protein activin A increased in parallel with hepcidin in serum from malaria-naive volunteers infected in controlled human malaria infection (CHMI) clinical trials. However, antibody-mediated neutralization of activin activity during murine malaria infection did not affect hepcidin expression, suggesting that these proteins do not stimulate hepcidin upregulation directly. In conclusion, we present evidence that the BMP/SMAD signaling pathway is perturbed in malaria infection but that activins, although raised in malaria infection, may not have a critical role in hepcidin upregulation in this setting.

## INTRODUCTION

Malaria is one of the world's deadliest and most geographically widespread human infectious diseases, causing hundreds of thousands of deaths per year (1). Malaria infections contribute significantly to the worldwide burden of anemia (2), and measures taken to decrease malaria at the population level frequently decrease anemia prevalence (3).

The mechanisms involved in the pathogenesis of malarial anemia include increased clearance of infected and uninfected red blood cells (iRBC and uRBC, respectively), dyserythropoiesis as a consequence of cytokine upregulation, and inadequate absorption and utilization of iron, for which the iron-regulatory hormone hepcidin has been implicated (reviewed in references 4–7). Hepcidin controls iron stores and localization by causing internalization and degradation of the iron export protein ferroportin (8), which mediates iron release into the circulation from erythrophagocytic macrophages and across the basolateral membrane of enterocytes (9–11). Raised hepcidin therefore inhibits both recycling of red cell iron through macrophages and iron absorption from the diet.

Hepcidin is upregulated during many bacterial, fungal, and viral infections (12–15) and also during symptomatic and asymptomatic natural human malaria infections (16–18), in volunteers undergoing controlled human malaria infection (CHMI) in clinical trials (19), and in malaria-infected mice (20, 21). Raised hepcidin during asymptomatic malaria infection is associated with poor iron absorption (22), and, in children with postmalarial anemia, with diminished erythrocyte incorporation of orally administered iron (23). Hepcidin renders mice infected with blood-stage malaria resistant to secondary sporozoite infection by decreasing the amount of iron in hepatocytes (20), and hepcidin-mediated macrophage iron sequestration has been proposed as a mechanism contributing to the increased growth of macrophage-tropic bacteria in malaria-infected hosts (24).

Hepcidin levels increase homeostatically under high-iron conditions (7) and in response to inflammation and infection (25) via the BMP/SMAD and interleukin-6 (IL-6)/STAT3 pathways, respectively. Appropriate regulation of hepcidin levels in response to fluctuations in iron is complex and requires many proteins, including Bmp6, HJV, Bmp type I and type II receptors, Hfe, and TfR2. These molecules combine to sense iron and to modulate transcription of hepcidin via the BMP/SMAD signaling pathway (26). In contrast, during anemia and under conditions of erythroid demand, hepcidin suppression occurs to facilitate iron release to plasma for erythropoiesis. A recently identified bone marrow-derived erythropoietin-induced hormone named erythroferrone likely plays a key role in hepcidin suppression in this context (27), which also appears to act via BMP/SMAD signaling (28).

The SMAD pathway also may be involved in the hepcidin response to inflammation, since the transforming growth factor β (TGFβ) superfamily member, activin B, is upregulated by inflammation in mice, associating it with hepcidin upregulation, independently of the IL-6 pathway (29). Activin B, a homodimer of two inhibin βB subunits, is known to be stimulated by inflammatory and infectious stimuli (30) and to contribute to hepcidin upregulation *in vitro* (29). However, it is unclear whether activin B is an essential component of the hepcidin response to inflammatory stimuli. One study noted that hepcidin upregulation in response to LPS was preserved in activin B knockout mice (*Inhbb*^−/−^) (31), suggesting that activin B is not required for hepcidin upregulation in this context, while a second noted that the activin binding protein follistatin blunted the hepcidin increase to lipopolysaccharide (LPS) in a murine model (32). Additionally, the roles of related proteins activin A (a homodimer of two inhibin βA subunits) and activin AB (a heterodimer of inhibin βB and inhibin βA subunits) are less defined with regard to hepcidin upregulation, with some studies (33) showing that activin A upregulates hepcidin but others demonstrating no effect of activin A or activin AB (32, 34).

Here, we study molecular regulation of hepcidin expression in the context of murine malaria infections and CHMI studies. We find evidence that the BMP/SMAD pathway is involved in hepcidin upregulation but that although activin B and activin A are increased in malaria, these molecules are unlikely to play a major role in controlling hepcidin expression.

## RESULTS

### Hepcidin upregulation during murine Plasmodium berghei infection is associated with increased BMP/SMAD pathway activity.

**FIG 1:**
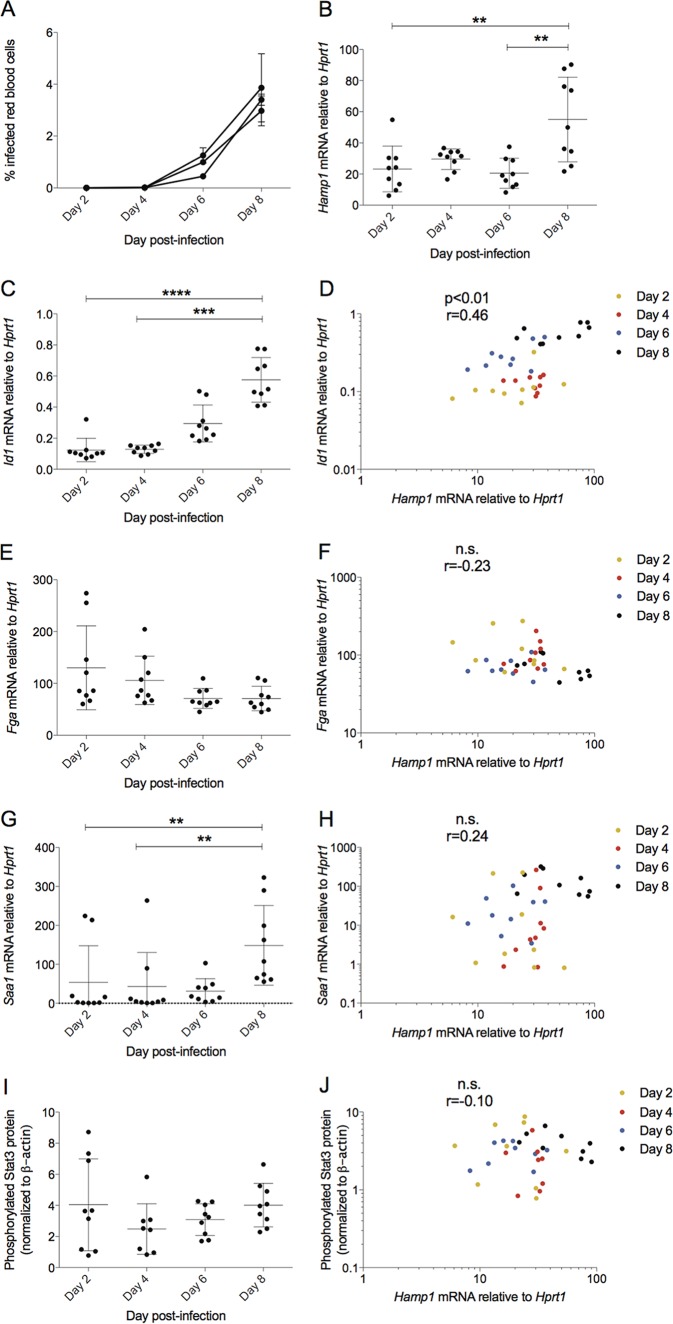
Hepcidin expression in Plasmodium berghei-infected BALB/c mice correlates with BMP pathway indicator gene *Id1*, not inflammatory pathway indicators. Mice were infected with P. berghei ANKA sporozoites, and groups were sacrificed at 2-day intervals postinfection. Data in all graphs are combined from 3 independent experiments (*n* = 3 mice/day/experiment, *n* = 9 total). (A) Mouse parasitemia as percent infected red blood cells, monitored by thin smear. (B) *Hamp1* mRNA in the liver increases on day 8 postinfection relative to day 2 (no parasitemia). BMP-responsive gene *Id1* increases on day 8 postinfection (C) and correlates significantly with hepcidin message (D). Acute-phase gene *Fga* message does not increase on day 8 postinfection (E) and does not correlate with hepcidin (F). Acute-phase gene *Saa-1* increases significantly on day 8 postinfection (G) but does not correlate with hepcidin (H). STAT3 phosphorylation does not increase significantly on day 8 postinfection (I) and does not correlate with hepcidin (J). All genes are shown as normalized to endogenous control gene *Hprt1*. Statistical analyses in dot plots are Dunn's multiple-comparison tests after Kruskal-Wallis test. **, *P* < 0.01; ***, *P* < 0.001; ****, *P* < 0.0001. In all correlation graphs, each symbol denotes a single mouse, and the color of the symbol indicates the day of sacrifice. All correlations are from Spearman's correlation tests. *P* values and *r* values are stated. ns, *P* > 0.05.

We infected male BALB/c mice with 10^3^ P. berghei ANKA sporozoites and harvested tissues from infected and control mice 2, 4, 6, or 8 days postinfection. Blood-stage parasitemia increased to 2 to 4% by 8 days postinfection (Fig. 1A). Hepatic hepcidin (*Hamp1*) mRNA expression was significantly increased on day 8 postinfection relative to day 2 (undetectable parasitemia) (Fig. 1B), consistent with previous studies showing elevated hepcidin mRNA only when parasitemia rises above a certain threshold (20, 21).

We then examined whether hepcidin expression was associated with the expression of genes indicative of activity of two well-characterized hepcidin regulatory pathways: the BMP/SMAD and IL-6/STAT3 pathways. We quantified hepatic expression of the BMP-responsive gene, inhibitor of DNA-binding 1 (*Id1*) (35), and the IL-6/STAT3-responsive acute-phase genes fibrinogen alpha (*Fga*) (12) and serum amyloid alpha-1 (*Saa-1*). *Id1* was significantly upregulated on day 8 postinfection relative to days 2 and 4 (Fig. 1C) and associated positively with *Hamp1* expression (Fig. 1D). This association remained significant when considering only the 9 mice from day 8 in the analysis (*P* = 0.05, *r* = 0.68) (Fig. 1D, black symbols). We also analyzed hepatic expression of three other BMP target genes, Atoh8, Smad6, and Smad7. *Atoh8* expression correlated with *Hamp1* expression overall (see Fig. S1 in the supplemental material) and when analyses were limited to day 8 (*P* < 0.01, *r* = 0.78); gene expression of *Smad6* and *Smad7* on day 8 also correlated with *Hamp1* (*P* < 0.01 for both, *r* = 0.73 and 0.75, respectively), although this correlation was not significant when including the earlier time points with lower parasitemia (Fig. S1).

Conversely, *Fga* expression was not increased on day 8 postinfection (Fig. 1E) and did not correlate with hepcidin (Fig. 1F). *Saa-1* increased on day 8 postinfection relative to days 2 and 4 (Fig. 1G) but also was not significantly correlated with hepcidin (Fig. 1H). We used quantitative Western blot detection to measure phosphorylated STAT3 (pSTAT3) directly: pSTAT3 was not significantly upregulated at day 8 postinfection relative to any time points (Fig. 1I, blots from representative experiment shown in Fig. S2) and did not correlate with hepcidin (Fig. 1J). When limiting analysis to day 8 samples (black symbols in all correlation graphs), there still was no significant association between *Hamp1* and *Fga*, *Hamp1* and *Saa-1*, or *Hamp1* and pSTAT3. These data suggest that in this blood-stage malaria model, increased BMP signaling parallels, and so may contribute to, *Hamp1* upregulation.

### Expression of activin B, not *Bmp* genes, increases in P. berghei infection.

**FIG 2:**
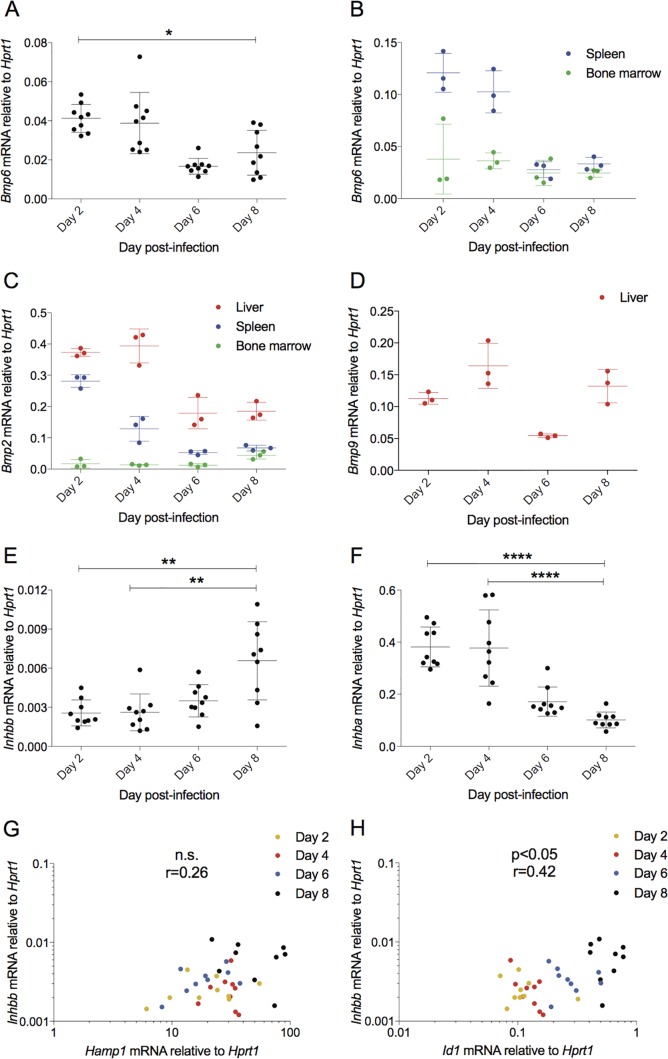
*Bmp* gene expression is not increased, but activin B (*Inhbb*) gene expression increases with hepcidin gene expression in Plasmodium berghei-infected BALB/c mice. (A) Liver *Bmp6* mRNA expression decreases on day 8 of infection (*n* = 3 mice per day per experiment, *n* = 9 total). (B to D) A representative experiment (*n* = 3 mice per day) was examined further to see if other *Bmp* genes increase in other candidate tissues. (B) *Bmp6* mRNA did not show any trend toward upregulation in spleen or bone marrow. (C) *Bmp2* did not increase in liver, spleen, or bone marrow. (D) *Bmp9* mRNA was undetectable in spleen and bone marrow and did not increase in liver. Hepatic activin B message (*Inhbb*) is increased on day 8 postinfection (E), while hepatic activin A mRNA (*Inhba*) is decreased (F). *Inhbb* expression shows a trend toward correlation with *Hamp1* (G) and correlates with *Id1* (H). Statistical analyses in dot plots are Dunn's multiple-comparison tests after Kruskal-Wallis test. *, *P* < 0.05; **, *P* < 0.01; ****, *P* < 0.0001. All correlations are from Spearman's correlation tests.

*Bmp6* knockout mice exhibit severe iron overload (36), and blocking Bmp6 *in vivo* also decreases hepcidin and increases serum iron (37). However, we found that hepatic *Bmp6* mRNA was downregulated as parasitemia increased (Fig. 2A). Other Bmp proteins are capable of stimulating hepcidin transcription *in vitro*, and Bmp2 has recently been shown to be a key hepatic regulator of hepcidin expression *in vivo* (38–41). We therefore examined whether *Bmp* genes were upregulated in the liver, bone marrow, and spleen samples. No significant increases in *Bmp6* (Fig. 2B) or *Bmp2* (Fig. 2C) mRNA were observed in any tissue on day 8 postinfection. *Bmp9* mRNA was undetectable in bone marrow and spleen and showed no increase on day 8 in the liver (Fig. 2D). Therefore, hepcidin and *Id1* upregulation during malaria infection was not accompanied by changes in expression of *Bmp* genes.

Recent reports have provided evidence that increased hepatic activin B expression during inflammation contributes to hepcidin induction, involving the BMP/SMAD pathway (29, 32). Activin A, closely related to activin B, is increased in animal and human sera following similar stimuli (30, 42). We found that hepatic activin B mRNA (*Inhbb*) was increased significantly on day 8 postinfection relative to days 2 and 4 (Fig. 2E), although activin A mRNA (*Inhba*) was significantly decreased (Fig. 2F). Activin B expression showed high intermouse variability but correlated significantly with the BMP response gene *Id1* (Fig. 2H), although it did not show significant correlation with *Hamp1* (Fig. 2G). Based on these results, we hypothesized that activin B contributes to hepcidin upregulation in murine blood-stage malaria infection.

### Activin B expression also increases at peak parasitemia during Plasmodium chabaudi infection.

**FIG 3:**
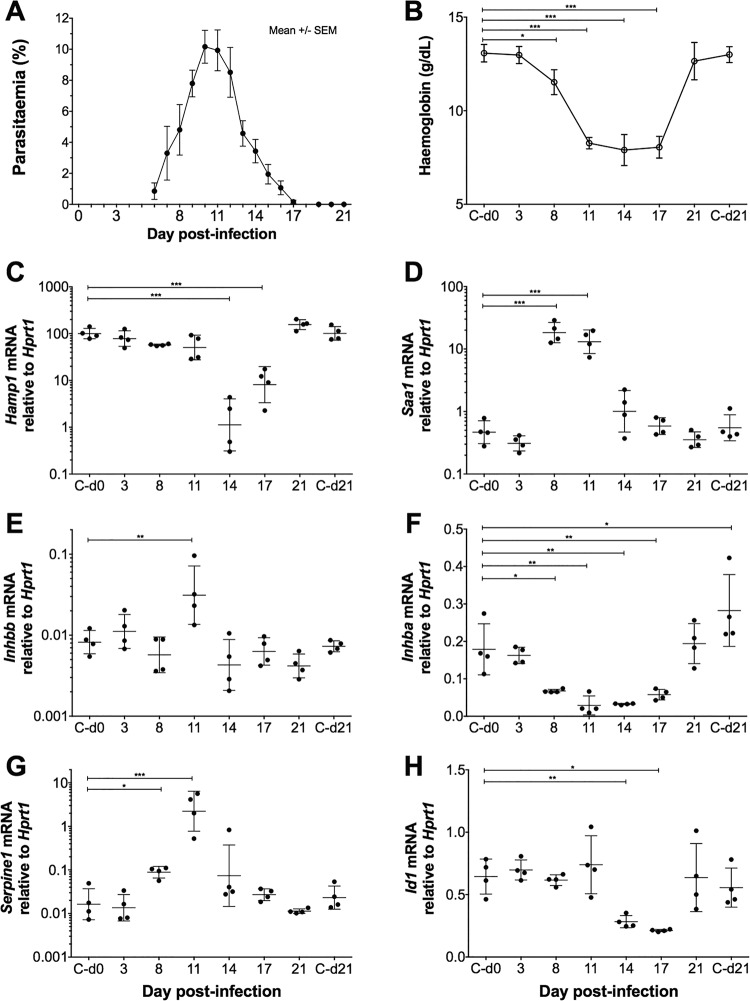
Changes in activin gene expression during the course of Plasmodium chabaudi infection of C57BL/6 mice. Groups of mice infected with PccAS were culled at intervals following infection (*n* = 4/group). (A) Mouse parasitemia as percent infected red blood cells (means ± standard errors of the means [SEM]), monitored by thin smear (films were made from each mouse each day until they were culled, and data are plotted from day 6). (B) Hemoglobin concentrations during PccAS infection (data are missing from one mouse on day 11 due to sample clotting); C-d0 and C-d21 represent data from uninfected control mice culled on day 0 and day 21. (C to G) Hepatic gene expression analysis by qRT-PCR, plotting expression relative to the endogenous control gene, *Hprt1*, yielding the following results: no detectable increase in *Hamp1* mRNA expression during parasitemia development but marked suppression during severe PccAS-associated anemia (C); upregulation of the acute-phase response gene *Saa1* as parasitemia increases (D); increase in *Inhbb* mRNA expression on day 11 postinfection (E); downregulation of *Inhba* around peak parasitemia (F); upregulation of activin-responsive gene *Serpine1* around peak parasitemia (G); BMP signaling indicator gene *Id1* expression decreases at peak parasitemia (H). Statistical analyses were by one-way ANOVA with Dunnett's multiple-comparison test (relative to controls on day 0), and posttests were used. *, *P* < 0.05; **, *P* < 0.01; ***, *P* < 0.001. Plots depict means ± standard deviations, except where *y* axes are plotted on log scales, in which case statistical analyses are performed on log-transformed data and plots show geometric means ± geometric standard deviations.

To investigate whether these data were more broadly applicable, we examined C57BL/6 mice infected with a second malaria parasite species, *Plasmodium chabaudi chabaudi* AS (PccAS), widely used as a self-resolving model of severe malarial anemia (43). In this experiment, C57BL/6 mice were injected intravenously with 10^5^ PccAS-infected erythrocytes, and groups of mice were culled at intervals following injection. Infected mice developed parasitemias that peaked around day 11 and then resolved (Fig. 3A). Severe anemia, demonstrated by marked reductions in hemoglobin concentration as parasitemia developed, reached a nadir concurrently with peak parasitemia and persisted for a further week before returning to normal (Fig. 3B). Regulation of hepcidin in this context is likely complicated due to conflicting signals during the concurrent inflammation and anemia, which enhance and suppress hepcidin expression, respectively. We did not observe increased liver *Hamp1* mRNA expression in the earlier phases of infection (Fig. 3C) despite evidence of inflammation (Fig. 3D, increased Saa1), likely because of signals that suppress hepcidin arising as anemia develops (for example, erythroferrone); indeed, as anemia becomes more severe, *Hamp1* expression is strongly decreased. Changes in activin gene expression similar to those observed in P. berghei infection were observed during escalation of this parasitemia: there was a significant upregulation of liver *Inhbb* mRNA expression at peak parasitemia (Fig. 3E), with a concomitant smaller decrease in liver *Inhba* expression (Fig. 3F). Consistent with activin signaling, there was increased expression of *Serpine1* (known to be induced by activin signaling) (Fig. 3G) as the parasitemia escalated to its peak. Paralleling *Hamp1* expression, hepatic *Id1* was not upregulated in early infection but was decreased as anemia became more severe (Fig. 3H), consistent with erythroid-mediated suppression of hepcidin (as a result of anemia) requiring a decrease in Smad signaling (28).

### Hepcidin and activin A peptide are upregulated in human volunteers experimentally infected with malaria.

Given these observed changes in activin expression in murine malaria infection, we sought to assess the applicability of our findings to human infection. We measured serum concentrations of hepcidin, activin A, C-reactive protein (CRP), and ferritin, besides transferrin saturation, in control subjects (*n* = 18) from three CHMI trials with P. falciparum. At the time of experimentation, we were unable to obtain serum assays for activin B. Although in the murine model hepatic activin A mRNA expression was suppressed at day 8, previous studies have suggested that hepatic activin A mRNA does not associate with serum activin A concentrations (30, 42).

Parasitemia data for each trial are shown Fig. S3A to C. Between trials, there were no differences in times from challenge (C) to day of diagnosis (DoD), from C to the first quantifiable quantitative PCR (qPCR) measurement postinfection (termed PCR patency), or in parasitemia measured by qPCR at DoD (Fig. S3D to F). In subsequent analyses, we therefore combined data from the three trials to increase power.

**FIG 4:**
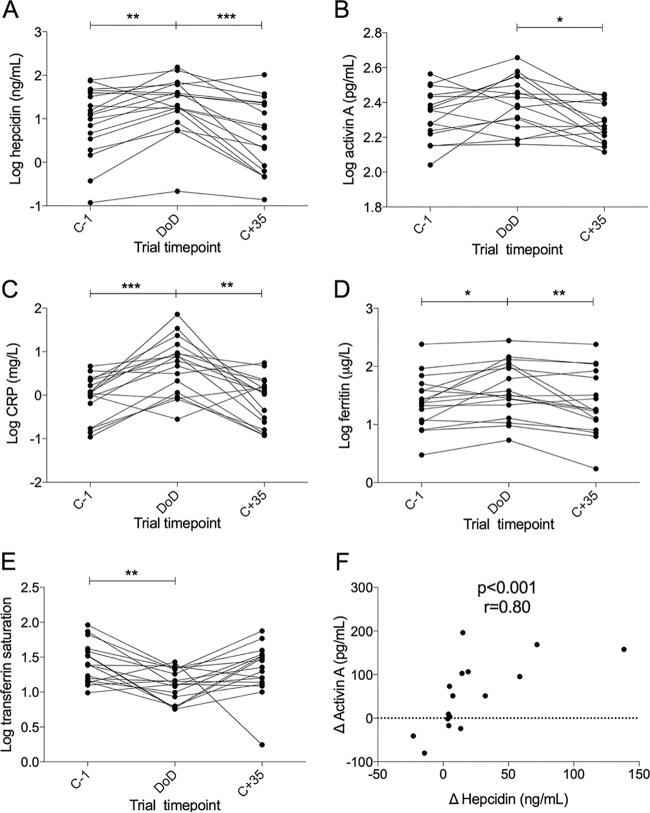
Changes in serum hepcidin, activin A C-reactive protein (CRP), ferritin, and transferrin saturation during CHMI trials. (A) Serum hepcidin increased on DoD compared to that at either the C−1 or C+35 time point. Hepcidin was measured in *n* = 18 volunteers. (B) Activin A was significantly upregulated at DoD versus the level at C+35. CRP (C) and ferritin (D) increased at DoD over other time points. (E) Transferrin saturation was reduced on DoD compared to that at C−1 only. (F) Δ serum hepcidin and Δ activin A were significantly correlated (Spearman's correlation test); each symbol denotes a single individual. Δ protein increases during infection were calculated by the value at DoD minus the mean of values at C−1 and C+35. Data were missing for iron and activin A measurements due to insufficient sample volume available (*n* = 17 in panels C, D, and E and *n* = 16 in panels B and F). (A to E) All data are log transformed, and statistical analyses are on log-transformed data. Statistical comparisons in before-after dot plots are Dunnett's multiple-comparison tests after ANOVA test. Spearman correlation is shown in panel F, and data are not log transformed. *, *P* < 0.05; **, *P* < 0.01; ***, *P* < 0.001.

Serum samples were taken 1 day prior to challenge (C−1), at DoD, and 35 days postinfection, when the infection had resolved (C+35). Hepcidin concentrations were significantly increased at DoD compared to those at both C−1 and C+35 (Fig. 4A). Activin A showed a trend toward an increase on DoD over C−1 and was significantly lower at the resolution of infection at C+35 (Fig. 4B). CRP (Fig. 4C) and ferritin (Fig. 4D) were elevated at DoD. Accordingly, transferrin saturation was decreased at DoD compared to that at C−1, indicative of inflammatory hypoferremia (Fig. 4E).

A strong correlation was evident between Δ hepcidin and Δ activin A protein in infection (where Δ is the value at DoD minus the average of values at C−1 and C+35). Those volunteers who demonstrated the most pronounced hepcidin increases at DoD also showed the greatest activin A increases (Fig. 4F). Full data for each volunteer are shown in Fig. S4.

In two published studies that compared activin A tissue mRNA levels with circulating protein, one tested only liver and the other examined multiple tissues, including spleen and bone marrow; neither found significant correlations (30, 42). Further work has suggested that stored activin A protein can be produced and released into the circulation by hematopoietic cells, and activin A can also be produced *de novo* from circulating white blood cells (44, 45). We previously demonstrated that hepcidin mRNA was upregulated in peripheral blood mononuclear cells (PBMC) from healthy malaria-naive donors cocultured with P. falciparum iRBC (46). We quantified activin A mRNA (*INHBA*) on samples from four donors from this study and found that both hepcidin (*HAMP*) and activin A mRNA were significantly upregulated in PBMC cocultured with iRBC but not uRBC (Fig. S5A and B). Thus, serum activin A induction during malaria infection may at least in part be produced by PBMC exposed to iRBC.

### Activin proteins upregulate hepcidin.

Previous studies have demonstrated hepcidin induction by recombinant activin B protein in primary hepatocytes or hepatoma cell lines (29, 32). Likewise, we found that activin B and, to a lesser extent, activin A, induced *HAMP* mRNA in HepG2 hepatoma cells at 4 h posttreatment (Fig. S6A) but did not significantly induce *ID1* mRNA (Fig. S6B). Hepcidin induction by both activin proteins was most pronounced between 1 and 8 h posttreatment (Fig. S6C and D). Pretreatment of HepG2 cells with the BMP type 1 receptor inhibitor molecule LDN-193189 (LDN) (47) prior to activin administration reduced the basal expression of both *HAMP* (Fig. S6E) and *ID1* (Fig. S6F) but did not decrease the proportional increase in response to activin treatment. These data are consistent with recent reports suggesting that activin A/B-mediated hepcidin upregulation proteins occur through the LDN-sensitive BMP type 1 receptor Alk2 or Alk3 (29, 32), but that this pathway is resistant to LDN inhibition as a result of Alk2 complex formation with ActRIIA (34).

### Anti-activin A/B antibodies do not decrease hepcidin expression in a malaria-infected mouse model.

**FIG 5:**
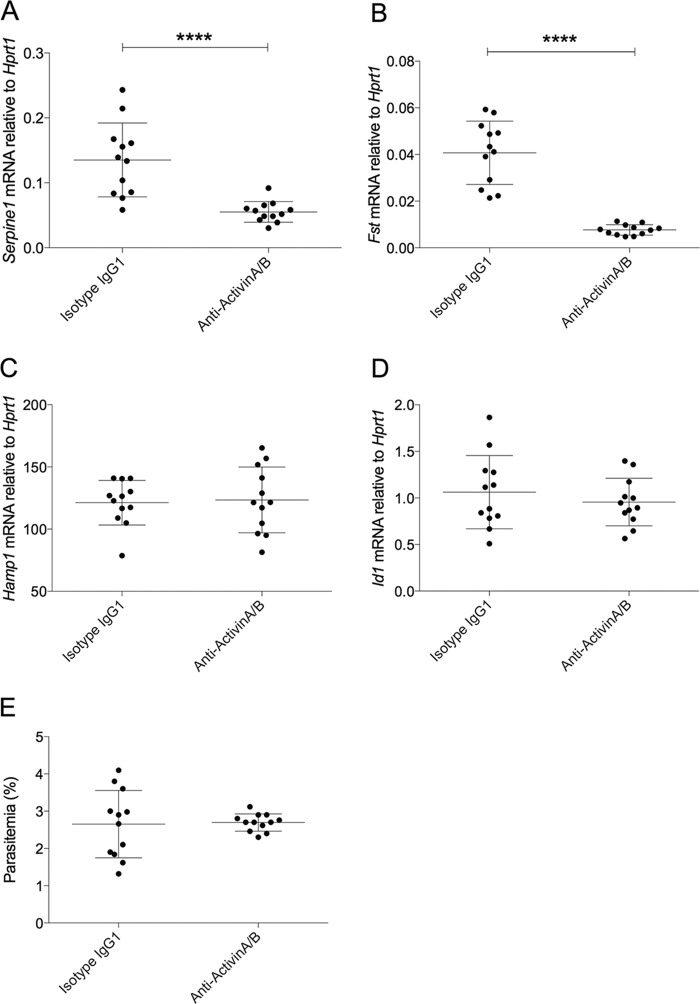
Hepcidin expression in murine P. berghei infection was not decreased by administration of anti-activin A and anti-activin B antibodies. Mice were infected with P. berghei ANKA sporozoites as described in the text and sacrificed at day 8 postinfection. Mice were injected i.p. twice with a cocktail of anti-activin A and anti-activin B antibodies (containing 100 μg of each, in 400 μl) at 6 and 30 h prior to sacrifice; controls were given isotype IgG control (200 μg, in 400 μl) only. Data in all graphs are combined from 2 independent experiments (*n* = 6 mice per treatment per experiment, *n* = 12 total). Gene expression is shown relative to housekeeping gene *Hprt1*. mRNA of activin-responsive genes *Serpine1* (A) and *Fst* (follistatin) (B) were significantly decreased in anti-activin treated mice, indicating antibody efficacy. *Hamp1* (C) and *Id1* (D) mRNA were not significantly altered between infected mice treated with anti-activin A/B and isotype IgG control. (E) Parasitemia on day 8 (day of sacrifice) was unchanged between groups. Statistical analyses are Mann-Whitney tests. ****, *P* < 0.0001.

We finally investigated whether inhibiting activin activity affected hepcidin expression during murine blood-stage P. berghei infection. Sporozoite-infected mice were treated with anti-activin A/B or isotype control antibodies on days 6 and 7 postinfection (30 and 6 h prior to sacrifice on day 8, respectively). Expression of *Serpine1* (encoding plasminogen activator inhibitor 1 [PAI-1]), which is responsive to activins (48) and the activin-regulatory gene encoding follistatin (*Fst*), were suppressed in mice treated with anti-activin antibodies, indicative of effective inhibition of liver activin signaling (Fig. 5A and B). However, *Hamp1* (Fig. 5C) and *Id1* expression (Fig. 5D) were not significantly different between malaria-infected mice treated with anti-activin A/B and isotype control antibodies. There was no difference in parasitemia between the two groups (Fig. 5E).

## DISCUSSION

Iron is required for Plasmodium growth, and host iron-handling proteins can influence outcome of infection; for example, lipocalin-2, which can sequester iron, controls the severity of Plasmodium yoelii infection in mice (49). Here, we focused on regulation of the iron-regulatory hormone hepcidin during malaria. Hepcidin is raised in uncomplicated malaria infection in mice and humans (18–20). Hepcidin redistributes iron into the macrophage compartment and away from the serum and so may contribute to dyserythropoiesis and the development of malarial anemia. In established severe malarial anemia, hepcidin then is suppressed (50) through a pathway that likely involves the erythroid progenitor-derived factor erythroferrone (51). The initial hepcidin upregulation blocks oral iron absorption during and after infection (22, 23) and plays a role in determining host susceptibility to malaria superinfection (20) and, possibly, coinfections with other pathogens (24). Understanding the mechanisms of hepcidin induction during uncomplicated malaria therefore is important.

IL-6 upregulates hepcidin and this cytokine increases in malaria infection, and in some studies, IL-6 has been correlated with hepcidin in human serum (50, 52, 53); however, in one study of infected children, urinary IL-6 and hepcidin were not associated (16). Murine studies have also shown different outcomes: one study found a close correlation between pSTAT3 levels and *Hamp1* (21), but another noted only minor IL-6 upregulation, without pSTAT3 increase, in infected mice (51). Hepcidin induction in primary hepatocytes treated with serum from infected mice was shown to be abrogated by the BMP pathway inhibitor dorsomorphin, while IL-6 neutralizing antibodies were less effective (20). Finally, PBMC, when cocultured with P. falciparum-infected red blood cells, upregulated hepcidin expression without appreciable IL-6 increases (46). The role of IL-6 in hepcidin upregulation in malaria infection is unclear.

During blood-stage P. berghei ANKA malaria infection in mice, we found that hepcidin expression correlated most closely with hepatic expression of the BMP/SMAD response genes *Id1* and *Atoh8* and less so with IL-6/STAT3 pathway response gene *Fga* or *Saa-1* or STAT3 phosphorylation. These findings indicate that hepcidin upregulation in this model co-occurs with BMP/SMAD pathway signaling. The expression of *Bmp* genes in infected mice was unchanged or moderately downregulated as parasitemia increased. Of relevance, previous work demonstrated that *Bmp* gene expression was not elevated in mice injected with LPS despite evidence of BMP/SMAD pathway activity occurring concurrently with *Hamp1* elevation (29). Hepatic activin B mRNA (*Inhbb*) was significantly raised post-LPS injection, suggesting that activins play a role in hepcidin upregulation through the BMP/SMAD pathway in inflammatory contexts (29, 30, 34). A previous study noted a marginal increase in hepatic message levels of activin B, and a decrease in *Bmp6*, in a murine model of malaria infection (51).

Activin proteins were initially discovered as reproductive factors, but increasing evidence has demonstrated a role in the acute host response to infectious and inflammatory stimuli, as well as shaping the subsequent immune response (54). Activin A increases in serum of septic human patients (55, 56) and in animals following LPS injection (30, 34, 42, 57). Notably, although activin A protein levels increase in sera post-LPS injection, hepatic expression of *Inhba* mRNA decreases (30, 42). This disconnect between activin A serum protein and liver expression have led researchers to hypothesize that activin A is at least partially regulated at the posttranscriptional level and/or produced by tissues other than the liver, such as bone marrow-derived cells (42, 58), peripheral blood monocytes (45), or dendritic cells (44). Activin B previously has been thought to be functionally similar to activin A, possibly with slightly weaker effects (59–61), although studies of knockout mice have identified some differences in the roles of the two proteins (62–64). The effect of activin A and activin B on hepcidin regulation has been debated (32–34). In our *in vitro* system, we found some effects of activin A on hepcidin upregulation, although it was less potent than activin B.

We found increased hepatic *Inhbb* expression in P. berghei-infected mice, occurring concurrently with hepcidin and *Id1* expression increase. Hepatic activin A (*Inhba*) mRNA was decreased (as described in reference 29) as *Hamp1*, *Inhbb*, and parasitemia increased. Similarly, increased hepatic *Inhbb* expression and decreased *Inhba* expression were observed during the peak parasitemia of Plasmodium chabaudi infection of C57BL/6 mice, together with upregulation of *Serpine1* (consistent with activin signaling).

Importantly, an increase in *Inhbb* expression does not formally indicate an increase in activin B protein, as activin βB subunits can also combine with activin βA subunits to form activin AB protein. However, given the message decrease in *Inhba* in the liver, decreasing the local concentration of activin βA subunits, it is likely that the *Inhbb* increase we observed did result in an increase in circulating activin B and not activin AB.

An enzyme-linked immunosorbent assay (ELISA) for human activin B protein has been reported (65) but was not available at the time of experimentation. However, given the known independence of activin A liver mRNA and serum protein levels in animal models (30, 42), a possible role of activin A in hepcidin upregulation, and several studies that indicate that serum levels of activin A and B are coupregulated in different inflammatory and infectious states (66–68), we next chose to extend our studies by quantifying serum activin A and hepcidin concentrations from humans infected with P. falciparum as part of CHMI clinical trials. We found that hepcidin was increased during untreated blood-stage parasite infection. A previous CHMI study also reported increased hepcidin (19), although importantly this prior study only noted hepcidin increases subsequent to the initiation of antimalarial treatment, which likely induces transient inflammation due to release of parasite-derived material into the bloodstream. The increase in hepcidin we observed was accompanied by increases in acute-phase markers, decreased transferrin saturation, and increased serum activin A. Moreover, changes in activin A and hepcidin during infection were correlated, with the volunteers who exhibited the greatest hepcidin induction also demonstrating the most marked increases in activin A concentration. To our knowledge, this is the first report of activin A induction in the context of malaria infection and also the first to directly compare hepcidin and activin A levels in human serum. The moderate response we observed in volunteers infected in CHMI trials may be more pronounced in naturally infected individuals in the field, in whom parasitemia levels can greatly exceed the low parasitemia that is allowed in CHMI. We also showed that human PBMC, when cocultured with iRBC, upregulated activin A mRNA, providing one plausible physiological source for increased activin A serum levels during infection.

Finally, we treated P. berghei-infected mice with a combination of anti-activin A and anti-activin B antibodies to test whether activin neutralization could abrogate the hepcidin response. The antibody treatment was efficacious in blocking activin signaling, as shown by significant decreases in activin-regulated genes *Serpine1* and *Fst*, but there was no observed change in hepcidin or *Id1* expression. These data suggest activins are not required for hepcidin upregulation in this murine model of Plasmodium infection, although they do not rule out that activins could play a role in hepcidin regulation in malaria in humans. Our findings are consistent with a recent study demonstrating that *Inhbb*^−/−^ mice continued to display hepcidin upregulation in response to LPS challenge (31) but contrast with another study that demonstrated abrogation of hepcidin upregulation after LPS challenge by use of the activin-binding protein follistatin (32).

In conclusion, we provide evidence that hepcidin upregulation in uncomplicated blood-stage Plasmodium infection is correlated with BMP/SMAD pathway activity in the liver. However, despite showing perturbations to expression of activins A and B in mouse and human malaria and confirming that activins induce hepcidin expression, neutralization of activins during murine malaria infection did not affect hepcidin. This suggests other factors are responsible for hepcidin expression in this context.

## MATERIALS AND METHODS

### Plasmodium berghei sporozoite infections of BALB/c mice.

Six- to 8-week-old male BALB/c mice (Harlan, United Kingdom) were housed under specific-pathogen-free conditions with *ad libitum* access to standard chow (2018SX; Fe^2+^ content of ∼200 ppm; Harlan-Teklad). Mice were infected intravenously (i.v.; via tail vein) with 10^3^ Plasmodium berghei ANKA sporozoites (obtained from Anopheles stephensi mosquito salivary glands 21 days after feeding on blood containing infectious gametocytes) in 200 μl RPMI as previously described (69). Control mice were injected with 200 μl RPMI i.v. Mice were culled and samples harvested at 2, 4, 6, or 8 days postinfection. For activin neutralization experiments, mice were injected intraperitoneally (i.p.) on days 7 and 8 postinfection (30 h and 6 h prior to culling, respectively) with 100 μg each of anti-activin A (MAB3381; Bio-Techne, Abingdon, United Kingdom) and anti-activin B (MAB659; Bio-Techne) antibodies, or 200 μg isotype control IgG1 (MAB002; Bio-Techne), in 400 μl Dulbecco's phosphate-buffered saline (PBS) (Gibco) vehicle.

### Plasmodium chabaudi infections of C57BL/6 mice.

Six-week-old female C57BL/6 mice were infected intravenously with 10^5^ *Plasmodium chabaudi chabaudi* AS (PccAS)-infected red blood cells in Krebs glucose solution (PBS plus 1% glucose), harvested during the escalation of parasitemia from donor C57BL/6 mice infected from a frozen parasite stock (serially blood passaged rather than recently mosquito transmitted). Groups of 4 mice were culled at days 3, 8, 11, 14, 17, and 21 postinfection; 2 groups of 4 uninfected mice were culled as controls on day 0 and day 21. Parasitemia was assessed by counting Giemsa-stained thin films.

### Mouse sample harvest and storage.

Mice were given a lethal anesthetic injection and blood was extracted via cardiac puncture; serum was isolated using BD SST Microtainers (Bunzl Healthcare, London, United Kingdom), and spleens, livers, and right hind legs were collected. For Plasmodium chabaudi infections, blood was also taken into BD EDTA Microtainers for assessment of anemia.

Livers and spleen explants (approximately 2 mm^3^) were preserved in RNAlater (Qiagen, Crawley, United Kingdom) for RNA extraction. Liver was snap-frozen in liquid nitrogen for Western blot analysis. Bone marrow was aspirated from tibias and immediately lysed in 350 μl RLT buffer (Qiagen), homogenized using QIAshredders (Qiagen), and stored at −20°C for later RNA extraction.

### Mouse serum iron measurements.

Serum iron and unsaturated iron binding capacity (UIBC) of mouse sera were measured using the iron/total iron binding capacity (TIBC) reagent set (Pointe Scientific), scaling the recommended protocol to 96-well-plate format (6% volume) and reading absorbances (560 nm) on an Infinite M200 Pro Tecan microplate reader. TIBC was calculated as serum iron plus UIBC. Transferrin saturation (percent) was calculated by serum iron divided by TIBC times 100.

### Measurement of hemoglobin concentrations in mice.

Hemoglobin concentrations were measured in EDTA-blood using an ABX Pentra60 benchtop analyzer (Horiba).

### Hepatoma cell culture and activin protein treatment.

All *in vitro* experiments were performed in biological duplicate. HepG2 human hepatoma cells (ECACC) were cultured in minimal essential medium (MEM-alpha modification; Sigma) supplemented with 10% fetal calf serum (FCS; PAA Laboratories), 2 mM glutamine, 100 U/ml penicillin, and 0.1 mg/ml streptomycin (all Sigma). Cells were plated in a 12-well plate at 2 × 10^5^ cells/ml, 1 ml/well, allowed to adhere overnight, and starved for 5 h in MEM-alpha with 0.1% FCS prior to activin treatment. Cells were treated with recombinant activin A (50 ng/ml), activin B (50 ng/ml), or BMP9 (100 ng/ml; all Bio-Techne) for 4 h unless otherwise stated. LDN-193189 (LDN; 100 nM; Axon Medchem), when used, was added 30 min before administration of activins/BMP9.

### Human PBMC culture and treatment.

PBMC had been isolated using a Ficoll (GE Healthcare) gradient from the heparinized blood of consenting healthy adult donors according to the Weatherall Institute of Molecular Medicine local procedures, as described previously (12, 46). Cells (5 × 10^6^ cells/ml, 1 ml/well, 12-well plate) plated in RPMI 1640 media (10% FCS supplemented with 2 mM glutamine, 100 U/ml penicillin, and 0.1 mg/ml streptomycin; all Sigma) were cocultured with 10^7^ P. falciparum A4 strain schizont iRBC or an equivalent number of control uRBC for 3 h as previously reported (46). Gene expression data from these experiments previously were published elsewhere (46); here, we investigated changes in activin expression in the same samples.

### RNA extraction, cDNA synthesis, and quantitative real-time PCR (qRT-PCR).

RNA was extracted using RNeasy minikits (Qiagen) according to the manufacturer's protocol. Mouse spleen and liver samples from RNAlater were homogenized using a TissueRuptor (Qiagen). Cells cultured *in vitro* were homogenized using QIAshredders (Qiagen).

RNA concentrations were quantified using a NanoDrop ND-1000 spectrophotometer (Wilmington, DE, USA). cDNA was synthesized using the high-capacity RNA-to-cDNA kit (Applied Biosystems). Gene expression was quantified relative to endogenous control genes by qRT-PCR using TaqMan gene expression master mix and inventoried TaqMan assays (all Applied Biosystems) in 10-μl reaction mixtures in technical duplicate on a 7500Fast or QuantStudio7 instrument (Applied Biosystems), as previously described (12). Details of the inventoried TaqMan gene expression assays used are shown in Fig. S7 in the supplemental material.

### Western blotting.

Approximately ∼10-mg frozen murine liver explants were lysed on wet ice using a TissueRuptor in lysis buffer (50 mM Tris-HCl, pH 8, 150 mM NaCl, 5 mM EDTA, pH 8, 1% NP-40, and inhibitors of proteases and phosphatases [all Sigma]) as in previous studies (70). Protein concentration was assessed using the Thermo Scientific Pierce protein assay (Fischer Scientific); lysates were diluted to 20 μg protein/10 μl solution with 1/3-volume bromophenol blue loading buffer, run through 12% SDS separating gel, and blotted onto activated polyvinylidene difluoride (PVDF) membranes. Size comparison was provided by a Bio-Rad Precision Plus Protein all blue ladder (Bio-Rad).

Membranes were blocked in Odyssey blocking buffer (LI-COR Biosciences) for 1 h and incubated overnight with a combination of two primary antibodies: mouse anti-β-actin (1:10,000; antibody AC15; Sigma-Aldrich) and either rabbit anti-phosphorylated STAT3 (pSTAT3; 1:1,000; D3A7; Cell Signaling) or rabbit anti-STAT3 (1:3,000; 79D7; Cell Signaling). Membranes were washed 3× in PBS–Tween (0.1%) and then incubated for 1 h with two secondary antibodies (donkey anti-mouse red 680 [1:20,000] and goat anti-rabbit green 800 [1:15,000]; LI-COR Biosciences) in 50% Odyssey–50% PBS buffer with 0.01% SDS and 0.1% Tween. Membranes were washed 3× in PBS–Tween (0.1%) and 1× in PBS only and then dried prior to examination with a LI-COR Biosciences instrument. Band intensities were quantified using LI-COR software. Each pSTAT3 or STAT3 band was normalized to its internal β-actin control prior to comparison with the mean of normalized band intensities of 3 uninfected age- and sex-matched mice sacrificed simultaneously and run on the same gel.

### CHMI studies.

Serum samples were obtained from eighteen 18- to 50-year-old, malaria-naive, unvaccinated volunteers from three separate United Kingdom CHMI clinical trials conducted to assess the efficacy of novel vaccines: NCT01623557, NCT00890760, and NCT01142765 (also termed VAC045, MAL034B, and VAC039, respectively), all registered with ClinicalTrials.gov (71–73). All volunteers gave written informed consent to participate and for their samples to be stored and used for further investigations to assess immunity to malaria. The samples analyzed presently were from the six nonvaccinated volunteers who formed the infectivity control group in each of the three CHMI studies.

As detailed elsewhere (71), five Anopheles stephensi mosquitoes infected with P. falciparum 3D7 clone sporozoites were allowed to bite each volunteer. The day of infection is termed the day of challenge (C). From day 6.5 postchallenge until 21 days postchallenge, volunteers were assessed once to twice daily by Giemsa-stained thick smear for the presence of parasites, and samples were collected for qRT-PCR analysis of P. falciparum parasitemia. Upon meeting the criteria for diagnosis (71–73), treatment with artemether-lumefantrine (Riamet) or atovaquone-proguanil (Malarone) was initiated. This time point was termed day of diagnosis (DoD) and also was typically the point of maximal parasitemia. Larger blood samples were collected the day before CHMI (C−1), at DoD, and after resolution of infection (C+35), and as such samples from these time points were available for investigation here.

### Measurement of human serum analytes.

Serum iron, UIBC, CRP, and ferritin in human samples were measured using an Abbott Architect cSystem Analyzer as described previously (14). TIBC and transferrin saturation were calculated as described above.

Serum hepcidin was quantified using the hepcidin-25 (human) enzyme immunoassay kit (EIA; Bachem), with the protocol modified as previously described (14, 74). Samples were initially measured at 1:8 dilution. Concentrations were determined using a 2-fold dilution curve (25 ng/ml to 0.05 ng/ml) as previously described (14). Duplicate concentrations with coefficients of variation of >15% were rerun. Samples with hepcidin concentrations falling outside the linear portion of the standard curve were rerun at appropriate dilutions. For those in which hepcidin was not detectable at the lowest possible dilution (typically 1:1), a value of 50% of the limit of detection (LOD; 0.118 ng/ml), multiplied by the dilution at which they were run, was assigned.

Serum activin A levels were measured by solid-phase sandwich ELISA (R&D Systems). Samples and standards were run in duplicate; samples with high coefficients of variation (>15%) were rerun.

### Statistical analysis.

Data processing was performed using Microsoft Excel. Statistical analyses were performed and graphs generated using GraphPad Prism (GraphPad Software, Inc., La Jolla, CA). All analyses on untransformed data were nonparametric. In mouse experiments, Kruskal-Wallis tests with Dunn's multiple-comparison posttests were used to specifically compare the group of interest with the others. In experiments with only two groups, Mann-Whitney tests were used. In human data with paired samples, data were log transformed and analysis of variance (ANOVA) with Dunnett's multiple-comparison tests were used. In grouped analyses shown in Fig. S5, ANOVA was used. All correlations are Spearman's correlations. The significance level was set at *P* = 0.05 throughout.

### Ethics.

All murine malaria infection experiments were performed in accordance with the terms of the United Kingdom Animals (Scientific Procedures) Act Project License (PPL 30/2889) and were approved by the University of Oxford Animal Welfare and Ethical Review Body.

All human CHMI trials were conducted in accordance with good clinical practices (GCP) and the principles of the Declaration of Helsinki. Trials were approved by the Oxfordshire Research Ethics Committee. The results of each trial and details of all necessary ethical and regulatory approvals for CHMI trials are reported elsewhere (71–73).

## Supplementary Material

Supplemental material
