# Supplementary material for: Role of Activins in Hepcidin Regulation during Malaria
Source: Infect Immun. 2017 Nov 17;85(12):e00191-17. doi: 10.1128/IAI.00191-17 (PMC5695100; doi:10.1128/IAI.00191-17)
Supplement: Supplemental material [file supp_85_12_e00191-17__index.html]

Supplemental material 

# Role of Activins in Hepcidin Regulation during Malaria

## Supplemental material

- Supplemental file 1 -

  Fig. S1. BMP response genes *Atoh8*, *Smad6*, and *Smad7* correlate with hepcidin during time point with highest parasitemia. Fig. S2. pStat3 and total Stat3 protein do not increase toward day 8 postinfection. Fig. S3. Parasitemia in three CHMI clinical trials. Fig. S4. Serum analytes measured in samples from CHMI trials. Fig. S5. PBMC upregulate HAMP and INHBA when cocultured with infected red blood cells but not uninfected RBC. Fig. S6. Recombinant activin A and activin B proteins upregulate HAMP and *ID1* mRNA *in vitro*. Fig. S7. Accession numbers for inventoried Taqman probes used for RT-PCR experiments.

  PDF, 1.4M
