# Supplementary material for: Role of Activins in Hepcidin Regulation during Malaria
Source: Infect Immun. 2017 Nov 17;85(12):e00191-17. doi: 10.1128/IAI.00191-17 (PMC5695100; doi:10.1128/IAI.00191-17)
Supplement: Supplemental material [file IAI.00191-17_zii999092211s1.pdf]

Spottiswoode et al

The role of activins in hepcidin regulation during malaria

Supplemental Material

Figure Legends

Fig. S1. BMP-response genes *Atoh8, Smad6, and Smad7* correlate with hepcidin during timepoint with highest parasitemia. mRNA from livers of mice in the experiments shown in Figures 1 and 2 was analysed for BMP response genes. Data in all graphs are combined from 3 independent experiments (*n*=3 mice/day/experiment, *n*=9 total). (A) *Atoh8* gene expression did not significantly increase during infection, but was significantly correlated with *Hamp1* (B). Hepatic *Smad6* mRNA (C) and *Smad7* mRNA (E) did not significantly change during infection and did not show a significant correlation with hepcidin message (D and F respectively); although both correlations become significant if analyses are restricted to day 8, when parasitemia was elevated. Statistical analyses in scatter plots are Dunn's multiple comparisons tests after Kruskal-Wallis test. In all correlation graphs, each symbol denotes a single mouse and colour of symbol indicates the day of sacrifice. All correlations are Spearman's correlation tests; p-values and r values are stated; n.s. denotes $p>0.05$.

Fig. S2. pStat3 and total Stat3 protein do not increase toward day 8 post-infection. pStat3 and Stat3 from mouse liver lysates were examined by Western blot. Images show all Western blots from a single representative sporozoite experiment. In all images, red bands at ~42 kilodaltons (kDa) are β-actin, green bands at ~80 kDa are pStat3 or total Stat3 as indicated. Green arrows indicate expected molecular weight of Stat3 or pStat3, red arrows indicate expected molecular weight of β-actin. Ladder marker sizes are indicated. Left three lanes of each blot are uninfected controls; right three lanes are liver lysates from infected mice.

Fig. S3. Parasitemia in three CHMI clinical trials. Control volunteers (*n*=6 per trial) were infected via five infectious mosquito bites at day of challenge. Thick smears were taken twice per day from day 6.5 post-challenge. Upon detection of parasites by microscopy (DoD), antimalarial treatment was initiated. Blood samples taken along with thick smears were analysed concurrently for precise parasitemia measurements using quantitative PCR. (A, B, C) show parasitemia as measured by qPCR in trials NCT01623557, NCT00890760, and NCT01142765, respectively. Each individual volunteer is depicted using a different colour. The three trials did not differ significantly in (D) days elapsing from challenge to diagnosis by microscopy, (E) days to parasitemia patency by qPCR, or (F) parasitemia at day of diagnosis (Kruskal-Wallis test, all $p>0.05$).

Fig. S4. Serum analytes measured in samples from CHMI trials. Serum concentrations of (A-C) hepcidin and (D-F) activin A in all three trials was variable at baseline and in degree of upregulation at DoD. Only a minority of volunteers showed major increases in (G-I) CRP and (J-L) ferritin at DoD. One volunteer in NCT01142765 (code number 14) exhibited very high ferritin throughout the study. (M-O) Transferrin saturation decreased at DoD in most volunteers.

Fig. S5. PBMC upregulate *HAMP* and *INHBA* when co-cultured with infected Red Blood Cells (iRBC) but not uninfected RBC (uRBC). We reanalysed cDNA from PBMC from four healthy malaria-naïve human donors that had previously been co-cultured with iRBC or uRBC for 3 h [42]. Both (A) *HAMP* and (B) *INHBA* mRNA were elevated in PBMC co-cultured with iRBC, but not those co-cultured with uRBC. Each donor's samples were run in biological and technical duplicate. Comparisons are Dunn's multiple comparisons tests after Friedman test. * $p<0.05$, ** $p<0.01$.

Fig. S6. Recombinant activin A and activin B proteins upregulate *HAMP* and *ID1* mRNA *in vitro*. Recombinant activin A or B protein was administered to hepatoma cells at 50 ng/mL for 4 h and the effects on (A) *HAMP* and (B) *ID1* shown. BMP9 (100 ng/mL) was included as a positive control. All gene expression shown is relative to housekeeping gene *GAPDH*. Each point shown depicts the average of two biological duplicates from an independent experiment

(*n*=4-6, not all conditions were run in each experiment). To better characterize the kinetics of activin response, HepG2 cells were treated as in (A-B) and harvested at timepoints ranging from 1 to 24 h post-treatment; maximal upregulation of (C) *HAMP* and (D) *ID1* was between 1-8 h post-treatment (*n* = 2 independent experiments, each experiment was run in biological and technical duplicate). To determine whether activins act via BMP receptors, LDN was added 30 minutes before activin or BMP9 application for 4 h. LDN inhibited (E) *HAMP* and (F) *ID1* upregulation by both activin proteins and by BMP9, but also decreased gene expression baseline (*n* = 3 independent experiments, each experiment was run in biological and technical duplicate). Statistical measures in dot plots (A-B) are Dunn's multiple comparison tests after Kruskal-Wallis test. Statistical measures in grouped bar graphs are Tukey's multiple comparison tests after 2-way ANOVA. ** $p<0.01$, ***$p<0.001$, ****$p<0.0001$.

Figure S7. Accession numbers for inventoried Taqman probes used for RT-PCR experiments. All probes were ordered from Life Technologies / ThermoFisher Scientific.

Supplementary Figure 1

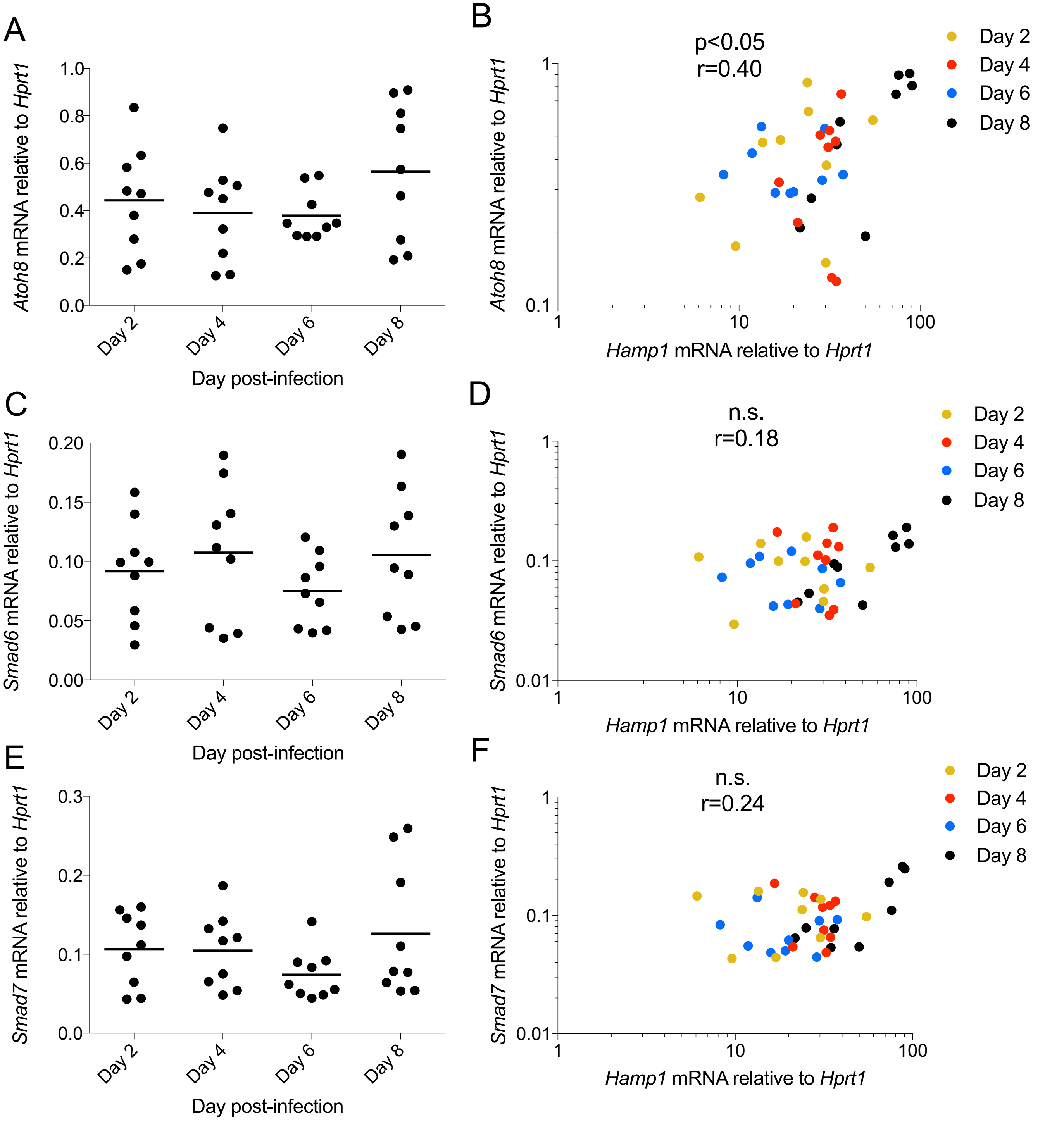

Fig. S1. BMP-response genes *Atoh8, Smad6, and Smad7* correlate with hepcidin during timepoint with highest parasitemia. mRNA from livers of mice in the experiments shown in Figures 1 and 2 was analysed for BMP response genes. Data in all graphs are combined from 3 independent experiments (*n*=3 mice/day/experiment, *n*=9 total). (A) *Atoh8* gene expression did not significantly increase during infection, but was significantly correlated with *Hamp1* (B). Hepatic *Smad6* mRNA (C) and *Smad7* mRNA (E) did not significantly change during infection and did not show a significant correlation with hepcidin message (D and F respectively); although both correlations become significant if analyses are restricted to day 8, when parasitemia was elevated. Statistical analyses in scatter plots are Dunn's multiple comparisons tests after Kruskal-Wallis test. In all correlation graphs, each symbol denotes a single mouse and colour of symbol indicates the day of sacrifice. All correlations are Spearman's correlation tests; p-values and r values are stated; n.s. denotes p>0.05.

Supplementary Figure 2

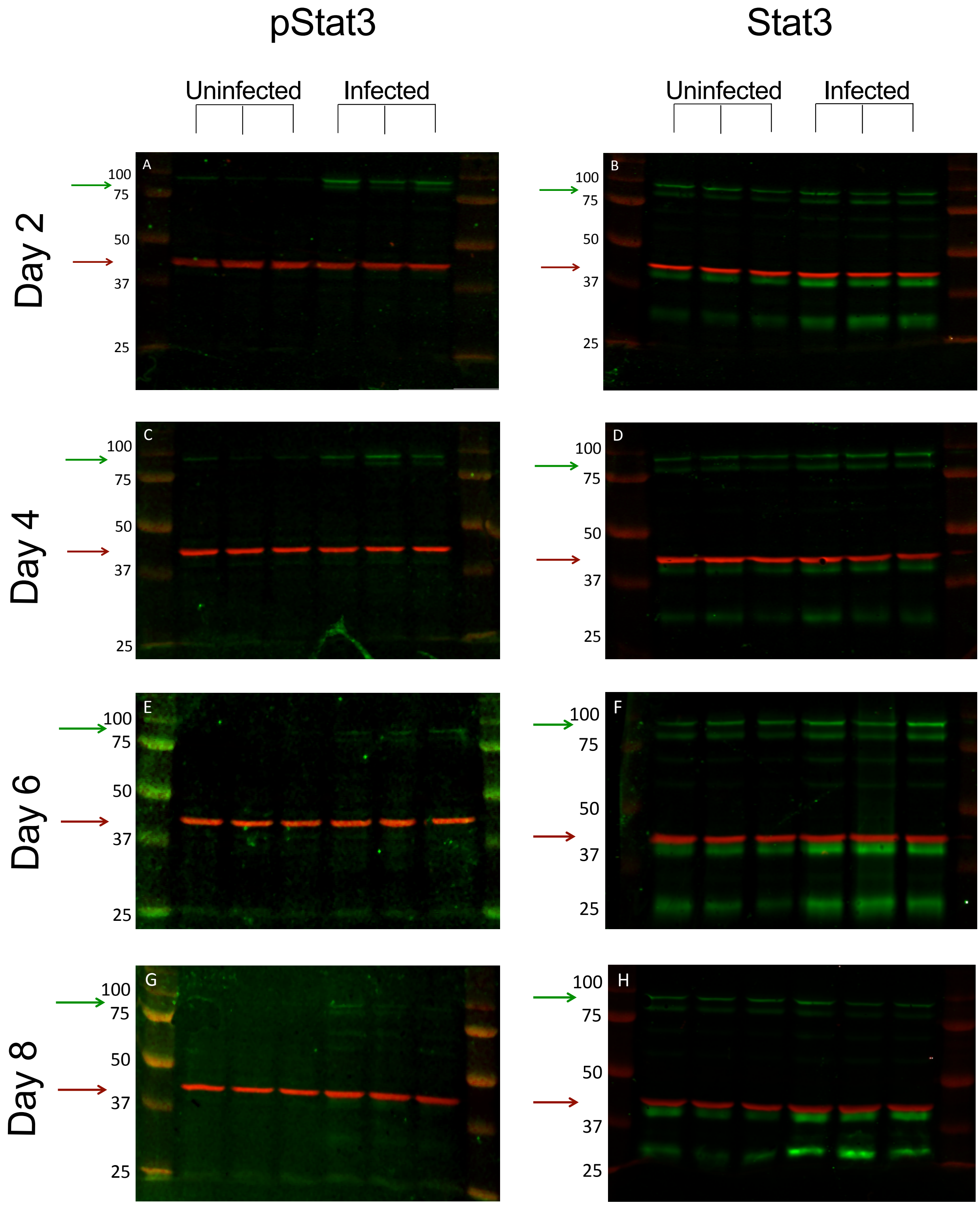

Fig. S2. pStat3 and total Stat3 protein do not increase toward day 8 post-infection. pStat3 and Stat3 from mouse liver lysates were examined by Western blot. Images show all Western blots from a single representative sporozoite experiment. In all images, red bands at ~42 kilodaltons (kDa) are β-actin, green bands at ~80 kDa are pStat3 or total Stat3 as indicated. Green arrows indicate expected molecular weight of Stat3 or pStat3, red arrows indicate expected molecular weight of β-actin. Ladder marker sizes are indicated. Left three lanes of each blot are uninfected controls; right three lanes are liver lysates from infected mice.

Supplementary Figure 3

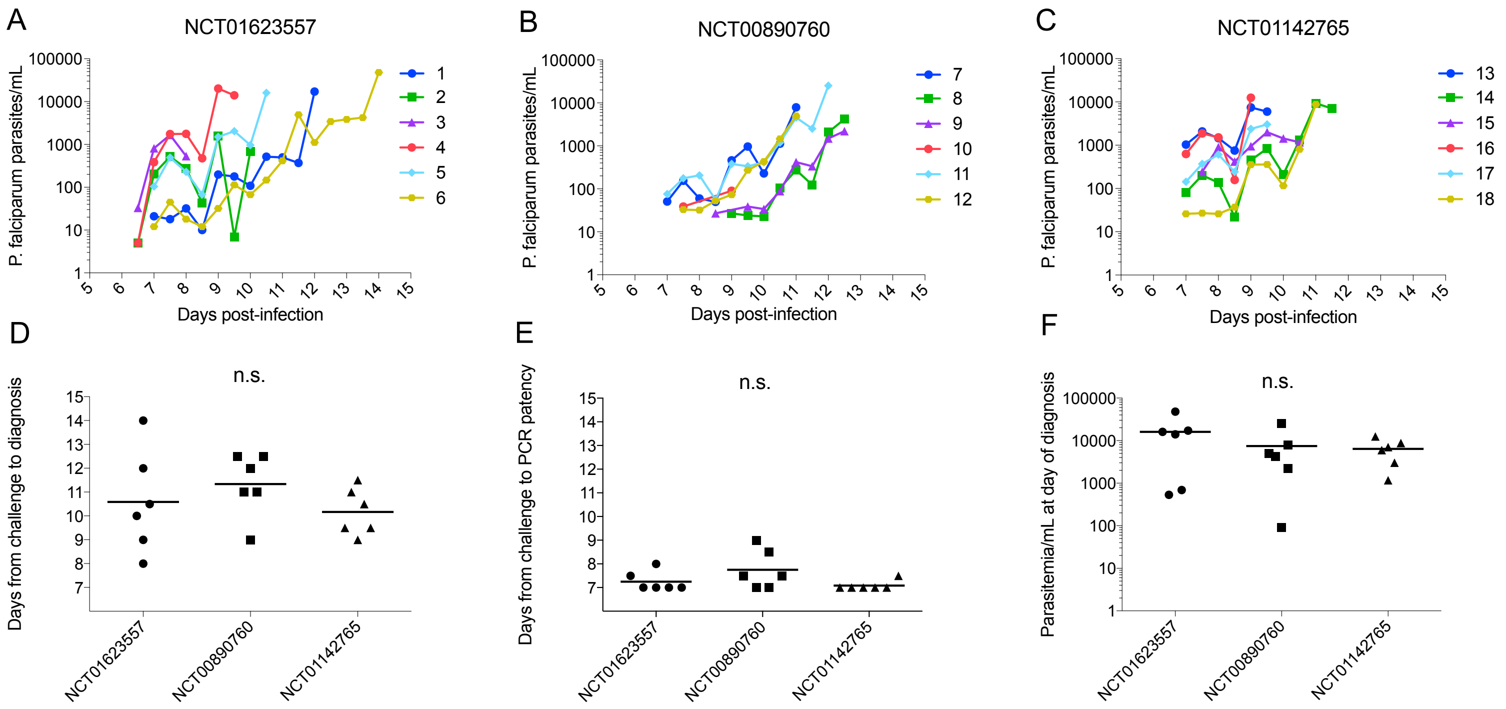

Fig. S3. Parasitemia in three CHMI clinical trials. Control volunteers (*n*=6 per trial) were infected via five infectious mosquito bites at day of challenge. Thick smears were taken twice per day from day 6.5 post-challenge. Upon detection of parasites by microscopy (DoD), antimalarial treatment was initiated. Blood samples taken along with thick smears were analysed concurrently for precise parasitemia measurements using quantitative PCR. (A, B, C) show parasitemia as measured by qPCR in trials NCT01623557, NCT00890760, and NCT01142765, respectively. Each individual volunteer is depicted using a different colour. The three trials did not differ significantly in (D) days elapsing from challenge to diagnosis by microscopy, (E) days to parasitemia patency by qPCR, or (F) parasitemia at day of diagnosis (Kruskal-Wallis test, all $p>0.05$).

Supplementary Figure 4

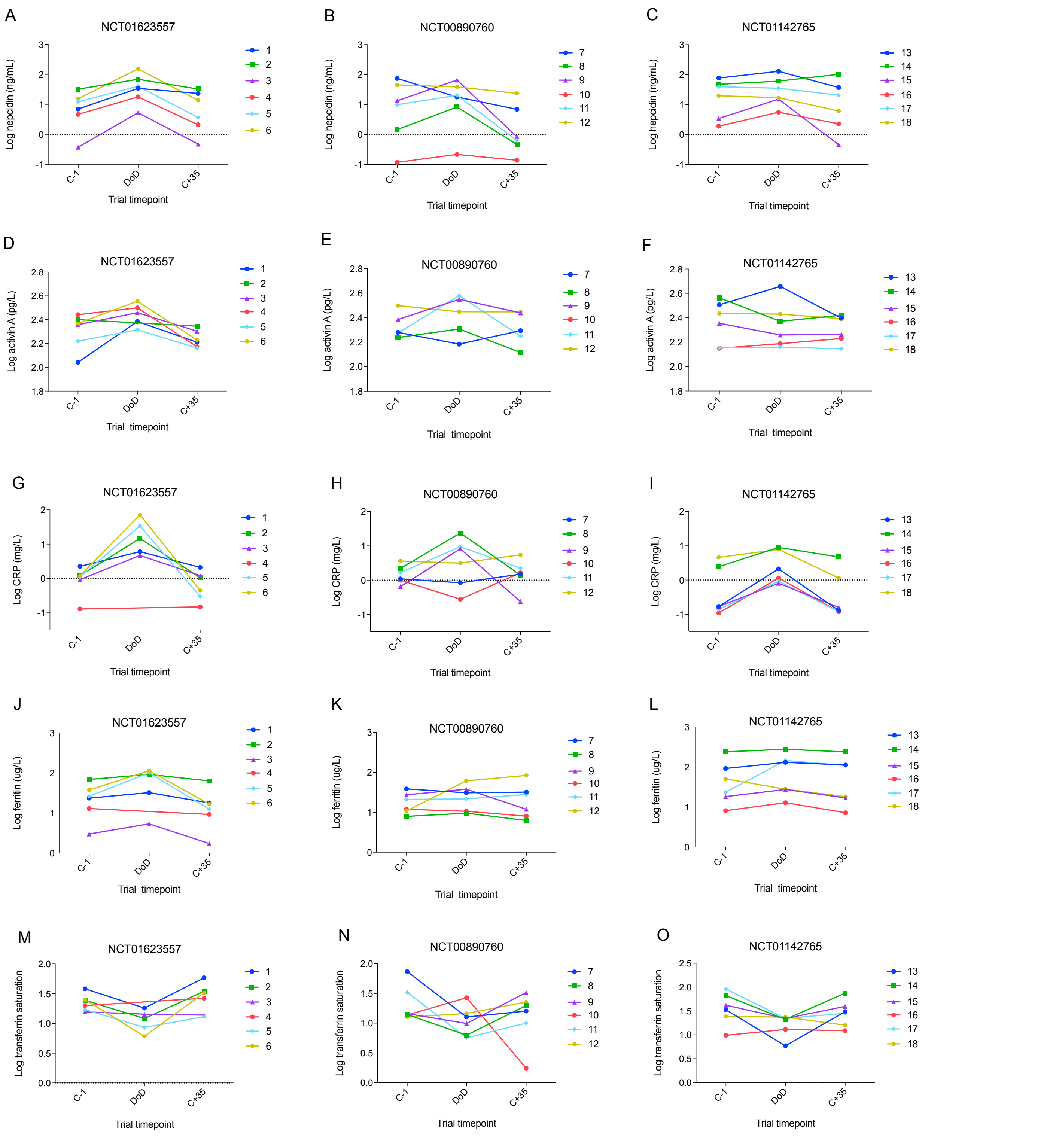

Fig. S4. Serum analytes measured in samples from CHMI trials. Serum concentrations of (A-C) hepcidin and (D-F) activin A in all three trials was variable at baseline and in degree of upregulation at DoD. Only a minority of volunteers showed major increases in (G-I) CRP and (J-L) ferritin at DoD. One volunteer in NCT01142765 (code number 14) exhibited very high ferritin throughout the study. (M-O) Transferrin saturation decreased at DoD in most volunteers.

Supplementary Figure 5

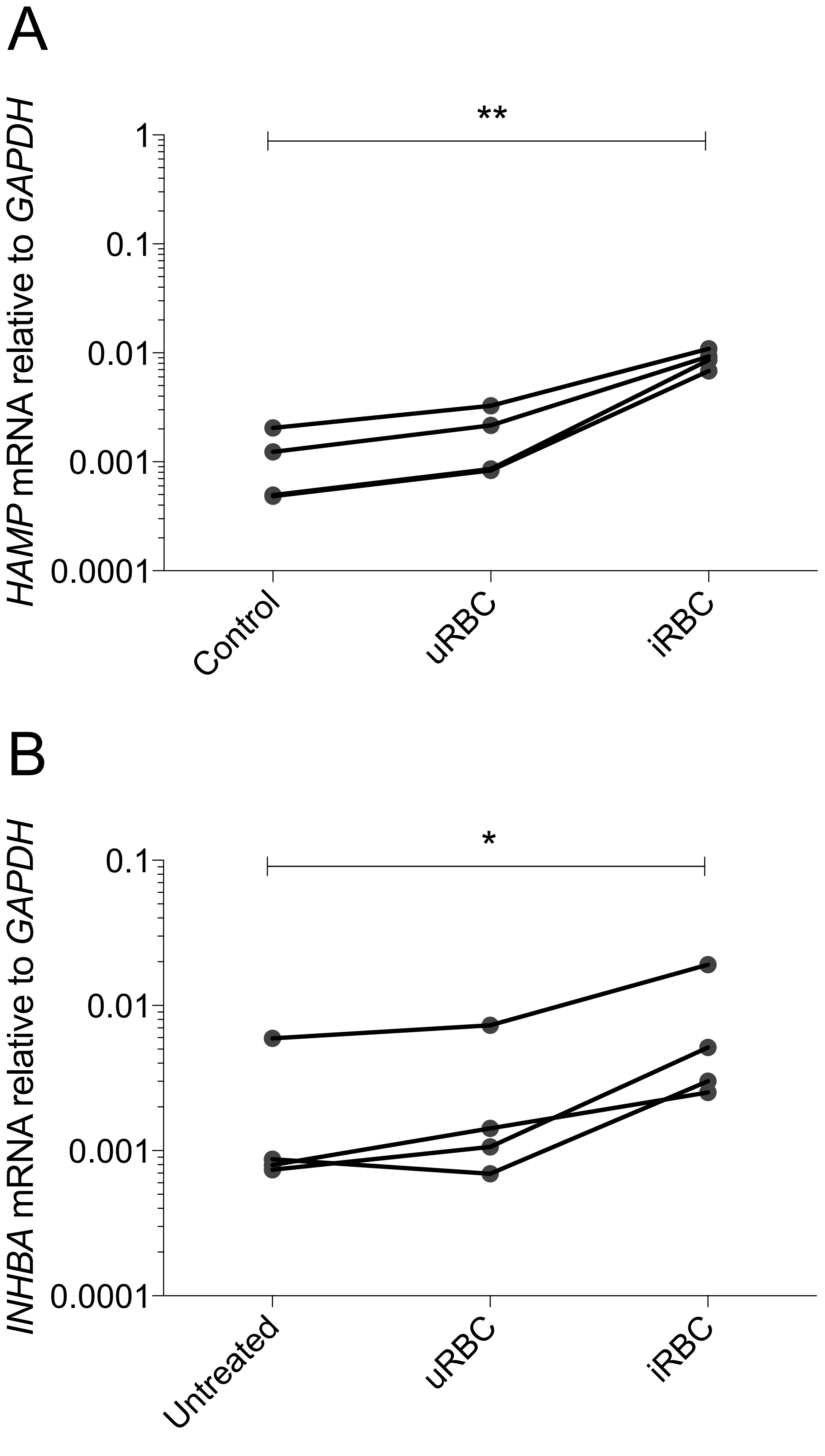

Fig. S5. PBMC upregulate *HAMP* and *INHBA* when co-cultured with infected Red Blood Cells (iRBC) but not uninfected RBC (uRBC). We reanalysed cDNA from PBMC from four healthy malaria-naïve human donors that had previously been co-cultured with iRBC or uRBC for 3 h [42]. Both (A) *HAMP* and (B) *INHBA* mRNA were elevated in PBMC co-cultured with iRBC, but not those co-cultured with uRBC. Each donor's samples were run in biological and technical duplicate. Comparisons are Dunn's multiple comparisons tests after Friedman test. * p<0.05, ** p<0.01.

Supplementary Figure 6

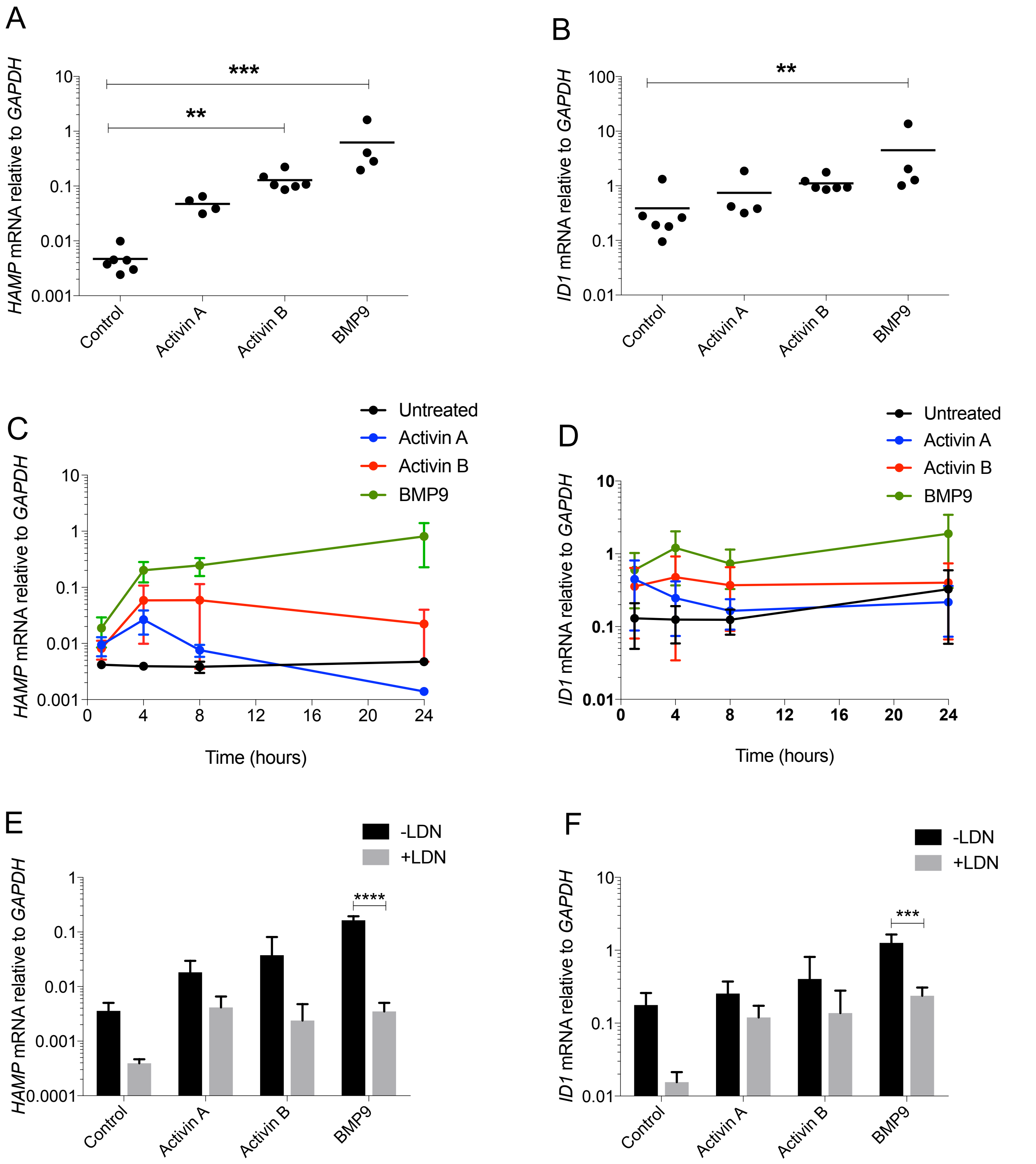

Fig. S6. Recombinant activin A and activin B proteins upregulate *HAMP* and *ID1* mRNA *in vitro*. Recombinant activin A or B protein was administered to hepatoma cells at 50 ng/mL for 4 h and the effects on (A) *HAMP* and (B) *ID1* shown. BMP9 (100 ng/mL) was included as a positive control. All gene expression shown is relative to housekeeping gene *GAPDH*. Each point shown depicts the average of two biological duplicates from an independent experiment (*n*=4-6, not all conditions were run in each experiment). To better characterize the kinetics of activin response, HepG2 cells were treated as in (A-B) and harvested at timepoints ranging from 1 to 24 h post-treatment; maximal upregulation of (C) *HAMP* and (D) *ID1* was between 1-8 h post-treatment (*n* = 2 independent experiments, each experiment was run in biological and technical duplicate). To determine whether activins act via BMP receptors, LDN was added 30 minutes before activin or BMP9 application for 4 h. LDN inhibited (E) *HAMP* and (F) *ID1* upregulation by both activin proteins and by BMP9, but also decreased gene expression baseline (*n* = 3 independent experiments, each experiment was run in biological and technical duplicate). Statistical measures in dot plots (A-B) are Dunn's multiple comparison tests after Kruskal-Wallis test. Statistical measures in grouped bar graphs are Tukey's multiple comparison tests after 2-way ANOVA. ** p<0.01, ***p<0.001, ****p<0.0001.

Supplementary Figure 7

Gene	Taqman probe
Mouse probes	
Hprt	Mm01545399_m1
Hamp	Mm00519025_m1
Id1	Mm00775963_g1
Fga	Mm00802584_m1
Saa1	Mm00656927_g1
Bmp6	Mm00432096_m1
Bmp2	Mm01340178_m1
Bmp9	Mm00807340_m1
Inhbb	Mm03023992_m1
Inhba	Mm00434339_m1
Serpine1	Mm00435858_m1
Fst	Mm00514982_m1
Atoh8	Mm00464055_m1
Smad6	Mm00484738_m1
Smad7	Mm00484742_m1

Human probes	
GAPDH	Hs02758991_g1
HAMP	Hs00221783_m1
ID1	Hs03676575_s1
INHBA	Hs03676575_s1

Figure S7. Accession numbers for inventoried Taqman probes used for RT-PCR experiments. All probes were ordered from Life Technologies / ThermoFisher Scientific.
